# Neoadjuvant checkpoint inhibitor immunotherapy for resectable mucosal melanoma

**DOI:** 10.3389/fonc.2022.1001150

**Published:** 2022-10-17

**Authors:** Joel Ho, Jane Mattei, Michael Tetzlaff, Michelle D. Williams, Michael A. Davies, Adi Diab, Isabella C. Glitza Oliva, Jennifer McQuade, Sapna P. Patel, Hussein Tawbi, Michael K. Wong, Sarah B. Fisher, Ehab Hanna, Emily Z. Keung, Merrick Ross, Roi Weiser, Shirley Y. Su, Michael Frumovitz, Larissa A. Meyer, Amir Jazaeri, Curtis A. Pettaway, B. Ashleigh Guadagnolo, Andrew J. Bishop, Devarati Mitra, Ahsan Farooqi, Roland Bassett, Silvana Faria, Priyadharsini Nagarajan, Rodabe N. Amaria

**Affiliations:** ^1^ Department of Melanoma Medical Oncology, The University of Texas MD Anderson Cancer Center, Houston, TX, United States; ^2^ Oncology Department, Hospital Moinhos de Vento, Porto Alegre, Brazil; ^3^ Division of Dermatopathology, University of California San Francisco (UCSF), San Francisco, CA, United States; ^4^ Department of Pathology, The University of Texas MD Anderson Cancer Center, Houston, TX, United States; ^5^ Department of Surgical Oncology, The University of Texas MD Anderson Cancer Center, Houston, TX, United States; ^6^ Department of Head and Neck Surgery, The University of Texas MD Anderson Cancer Center, Houston, TX, United States; ^7^ Department of Gynecologic Oncology and Reproductive Medicine, The University of Texas MD Anderson Cancer Center, Houston, TX, United States; ^8^ Department of Urologic Oncology, The University of Texas MD Anderson Cancer Center, Houston, TX, United States; ^9^ Department of Radiation Oncology, The University of Texas MD Anderson Cancer Center, Houston, TX, United States; ^10^ Department of Biostatistics, The University of Texas MD Anderson Cancer Center, Houston, TX, United States; ^11^ Department of Abdominal Imaging, Division of Diagnostic Imaging, The University of Texas MD Anderson Cancer Center, Houston, TX, United States

**Keywords:** mucosal melanoma, immunotherapy, melanoma, neoadjuvant, resectable

## Abstract

**Background:**

Neoadjuvant checkpoint inhibition (CPI) has recently demonstrated impressive outcomes in patients with stage 3 cutaneous melanoma. However, the safety, efficacy, and outcome of neoadjuvant CPI in patients with mucosal melanoma (MM) are not well studied as MM is a rare melanoma subtype. CPI such as combination nivolumab and ipilimumab achieves response rates of 37-43% in unresectable or metastatic MM but there is limited data regarding the efficacy of these agents in the preoperative setting. We hypothesize that neoadjuvant CPI is a safe and feasible approach for patients with resectable MM.

**Method:**

Under an institutionally approved protocol, we identified adult MM patients with resectable disease who received neoadjuvant anti-PD1 +/- anti-CTLA4 between 2015 to 2019 at our institution. Clinical information include age, gender, presence of nodal involvement or satellitosis, functional status, pre-treatment LDH, tumor mutation status, and treatment data was collected. Outcomes include event free survival (EFS), overall survival (OS), objective response rate (ORR), pathologic response rate (PRR), and grade ≥3 toxicities.

**Results:**

We identified 36 patients. Median age was 62; 58% were female. Seventy-eight percent of patients received anti-PD1 + anti-CTLA4. Node positive disease or satellite lesions was present at the time of treatment initiation in 47% of patients. Primary sites of disease were anorectal (53%), urogenital (25%), head and neck (17%), and esophageal (6%). A minority of patients did not undergo surgery due to complete response (n=3, 8%) and disease progression (n=6, 17%), respectively. With a median follow up of 37.9 months, the median EFS was 9.2 months with 3-year EFS rate of 29%. Median OS had not been reached and 3-year OS rate was 55%. ORR was 47% and PRR was 35%. EFS was significantly higher for patients with objective response and for patients with pathologic response. OS was significantly higher for patients with pathologic response. Grade 3 toxicities were reported in 39% of patients.

**Conclusion:**

Neoadjuvant CPI for resectable MM is a feasible approach with signs of efficacy and an acceptable safety profile. As there is currently no standard approach for resectable MM, this study supports further investigations using neoadjuvant therapy for these patients.

## Introduction

Mucosal melanoma (MM) is an aggressive form of melanoma that arises from melanocytes present on the mucosal lining of the respiratory, gastrointestinal and urogenital systems (1). MM differs from cutaneous melanoma because of its more aggressive biological behavior, lower tumor mutation burden, and a unique driver mutation profile (2, 3). This rare patient population is often excluded from melanoma clinical trials and there is limited prospective data to guide management of disease in both resectable and unresectable settings.

The 5-year overall survival rate for MM is about 25% with a significantly poorer survival compared to other subtypes of melanoma (4). This may be related to a more advanced disease at diagnosis and the challenges of achieving definitive local control at presentation due to anatomic constraints. Wide local excision of the MM with negative margins is associated with a better prognosis. However, given its high rates of local failure and development of distant metastasis, relapse-free survival is generally short (5, 6). Achieving a R0 surgical resection is especially challenging because of the multifocal pattern of growth, the complexities of the anatomical sites, and the higher proportions of patients who present with locally advanced disease and require extensive and potentially disfiguring surgeries (6, 7). Postoperative radiation therapy improves the rates of local control, but there is no evidence that it improves overall survival in MM patients (8). The magnitude of benefit of adjuvant systemic therapy is uncertain in MM and the utility of routine lymph node sampling and completion lymph node dissection is being investigated (9–12).

Neoadjuvant therapy is a very promising therapeutic approach in patients with clinical stage III cutaneous melanoma. Neoadjuvant immunotherapy possesses the advantage of immunological priming with the tumor cells *in situ* and earlier initiation of systemic therapy in these patients may be beneficial in delaying or preventing distant spread of disease (13). Neoadjuvant therapy may also potentially facilitate resection of disease by tumor downstaging before surgery. However, there are few studies investigating the role of neoadjuvant therapy in MM. A recent published neoadjuvant phase II trial with 21 MM patients who received neoadjuvant toripalimab/axitinib followed by adjuvant toripalimab reported a pathological response rate of 30% with a median recurrence-free survival (RFS) of 55.7 weeks (14).

As the neoadjuvant approach is not well studied in MM, we conducted this retrospective cohort study evaluating the clinical and pathologic responses and outcomes after neoadjuvant immunotherapy in patients with resectable MM.

## Materials and methods

Patients treated for MM between January 2015 to December 2019 were identified from our single-center institutional database at MD Anderson Cancer Center, Houston, Texas. We then extracted patients with resectable MM treated with neoadjuvant checkpoint inhibitors (Anti-CTLA4 and/or Anti-PD1). Clinical information including gender (male or female), age, LDH (above or below upper limit of normal), ECOG (Eastern Cooperative Oncology Group) performance status, primary tumor localization (head and neck, anorectal, urogenital and esophageal), nodal involvement and satellitosis were obtained. Additionally, the following pathologic features of the primary tumor were recorded: ulceration (present or absent); Breslow depth (tumor thickness); and mutation status (BRAF, cKIT, NRAS mutated or wild type).

Clinical outcomes assessed were overall survival (OS) defined as time from start of treatment date to date of death and event-free survival (EFS) defined as time from start of treatment date to date of first progression. Different starting times were used for different analyses. For analyses by treatment, the start date was the date of treatment. For analyses by objective response, the start date was the date of the assessment of objective response at 42 days after the start of treatment. For analyses by pathologic response, the start date was the date of surgery. For patients without surgery, the start date was assumed to be the same as the date of objective response assessment. For EFS, the event was the earlier of disease progression or death, and patients who remained alive without disease progression were censored at the last follow-up date. The same starting times were used for OS.

Objective response rate (ORR) was evaluated by a radiologist per RECIST 1.1 (15), and pathologic response rate (PRR) was evaluated by a pathologist according to the International Neoadjuvant Melanoma Consortium (INMC) criteria: pathologic complete response defined as absence of residual viable tumor and pathologic partial response defined as ≤ 50% of viable tumor cells (16). Clinical outcomes were assessed for all patients including patients who did not undergo surgery due to progression or complete response. The Kaplan-Meier method was used to estimate the distribution of OS and EFS and distributions were compared using the log-rank test. All statistical analyses were performed using R version 4.1.1. All statistical tests used a two-sided significance level of 5%. Treatment toxicities were categorized in Toxicities per Common Terminology Criteria for Adverse Events (CTCAE) version 5.

## Results

Thirty-six patients were identified in the database with resectable MM treated with neoadjuvant checkpoint inhibitors. Baseline characteristics of patients are described in **Table 1**. The median age of patients was 62 and 58% were female. Seventeen (47%) patients had nodal involvement or clinically or pathologically detected satellitosis. The most common primary site was anorectal (53%) followed by urogenital (25%), head and neck (17%), and esophageal (6%). Mutation analysis was not accessible for 3 patients. KIT mutation (30%) was the most common, followed by NRAS (12%) and BRAF (6%) mutations.

**Table 1:** Baseline characteristics of patients with mucosal melanoma treated with neoadjuvant checkpoint inhibitors.

Total number of patients	36	%
Median Age (range)	62 (33-83)	
Female patients	21	58
Primary site involvement only	19	53
Node positive or satellitosis	17	47
Satellitosis	3	8
ECOG 0-1	36	100
Ulceration	24	67
LDH > ULN	8	22
Mutations:		
KIT mutant	10	30
BRAF mutant	2	6
NRAS mutant	4	12
Not assessed	3	8
Primary site of disease:		
Anorectal	19	53
Urogenital	9	25
Head and Neck	6	17
Esophagus	2	6

ECOG, Eastern Cooperative Oncology Group; LDH, lactate dehydrogenase; ULN, upper limit of normal.

Treatment data was summarized in **Table 2** and **Supplemental Table 1**. All patients received neoadjuvant checkpoint inhibitors. Twenty-eight (78%) patients received combination anti-PD1 and anti-CTLA4. Anti-PD1 and anti-CTLA4 monotherapy was used in 7 (19%) and 1 (3%) patients, respectively. Seventeen (47%) patients received adjuvant systemic therapy. Sixteen (44%) and 1 (3%) patients received adjuvant Anti-PD1 and biochemotherapy, respectively (**Supplemental Table 2**). Nine (25%) patients received adjuvant radiation. Median time from last dose of neoadjuvant systemic therapy to surgery was 31 days.

**Table 2:** Neoadjuvant and adjuvant treatment received by patients with mucosal melanoma treated with neoadjuvant checkpoint inhibitors.

Total Number of Patients	36	%
Neoadjuvant Treatment Received	36	100
Anti-PD1 and anti-CTLA4	28	78
Anti-PD1 Monotherapy	7	19
Anti-CTLA4 Monotherapy	1	3
Adjuvant Treatment Received	17	47
Adjuvant anti-PD1	16	44
Adjuvant biochemotherapy	1	3
Adjuvant radiation	9	25

Twenty-seven (75%) of patients underwent surgery (**Supplemental Table 3**). Six patients (17%) had disease progression to unresectable disease prior to surgery. Five of these patients had combination Anti-PD1 and Anti-CTLA4 and 1 patient had Anti-CTLA4 only. Three patients (8%) had a complete objective response prior to surgery and did not undergo surgery; all remain disease-free at 35.5, 43.7, and 73.8 months following initiation of neoadjuvant systemic therapy. With a median follow-up of 37.9 months, the median EFS was 9.2 months and the 2-year and 3-year EFS were 36% and 29%. The 2-year OS was 64% and 3-year OS 55%; median OS had not been reached (**Table 3**). The ORR was 47% with 23% of patients having a complete response and 23% having a partial response (**Table 4**). PRR was 35%, with 26% of patients having pathologic complete or near complete response (**Table 5**).

**Table 3:** Event free survival (EFS), and overall survival (OS) of patients with mucosal melanoma treated with neoadjuvant checkpoint inhibitors.

Outcome	Median (95% CI)
2-year EFS (%)	35.7 (22.5 – 56.5)
3-year EFS (%)	28.5 (16.4 - 49.7)
Median EFS (months)	9.2 (7.1 – 30.1)
2-year OS (%)	64.2% (49.6 - 83.0)
3-year OS (%)	55.0% (39.4 - 76.9)
Median OS (months)	NR (12.1 – NR)

NR, not reached; CI, confidence interval.

**Table 4:** Objective response rate (ORR) of patients with mucosal melanoma treated with neoadjuvant checkpoint inhibitors.

Radiographic Response	#	%
Not accessible	6	
CR	7	23
PR	7	23
SD	6	20
PD	10	33
ORR	14	47

CR, complete response; PR, partial response; SD, stable disease; PD, progressive disease.

**Table 5:** Pathologic response rate (PRR) of patients with mucosal melanoma treated with neoadjuvant checkpoint inhibitors.

Pathological Response	#	%
Not accessible	5	
pCR or near pCR	8	26
pPR	3	10
pNR	20	65
pRR	11	35

pCR, pathologic complete response; pPR, pathologic partial response; pNR, no pathologic response.

**Figure 1:**
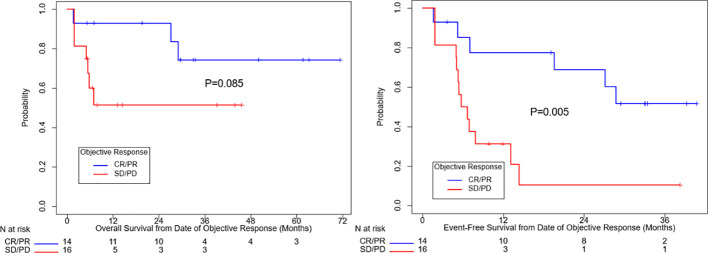
Overall survival and event free survival stratified based on objective response per RECIST v1.1. CR, complete response, PR, partial response, SD, stable disease, PD, progressive disease.

**Figure 1** summarizes OS and EFS by objective response. For OS, objective response was associated with improvement (HR=0.31, 95% CI: (0.078, 1.25), p=0.085). Estimates of OS at 3 years were 74% for patients with a complete or partial response and 51% for patients with no objective response. For EFS, the association with objective response was statistically significant (HR=0.24, 95% CI: (0.085, 0.69), p=0.005). Year 3 estimates of EFS were 52% for patients with a complete or partial response and 10% for patients with no objective response.

**Figure 2:**
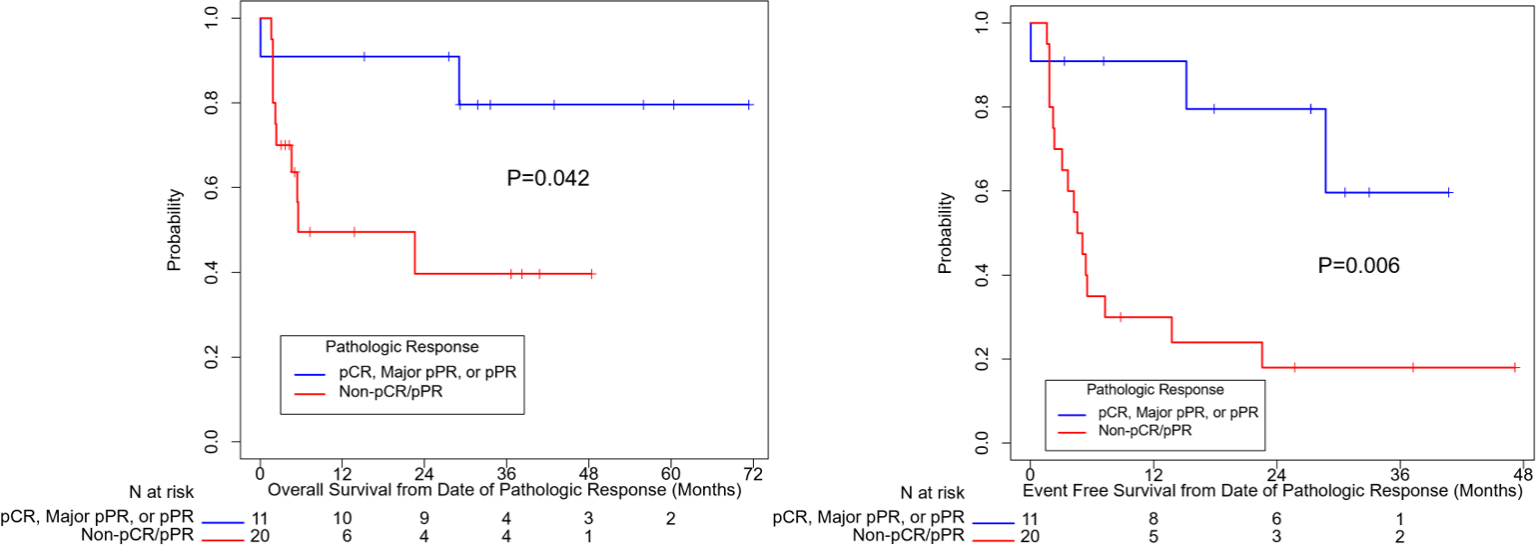
Overall survival and event free survival stratified based on pathologic response per INMC criteria. pCR, pathologic complete response; pPR, pathologic partial response.

**Figure 2** summarizes OS and EFS by pathologic response. Pathologic response was associated with a significant improvement in OS (HR=0.23, 95% CI: (0.048, 1.07), p=0.042). Estimates of OS at 3 years were 80% for patients with a complete, major, or partial pathologic response and 40% for patients with no pathologic response. For EFS, the association with pathologic response was also statistically significant (HR=0.20, 95% CI: (0.058, 0.71), p=0.006). Year 3 estimates of EFS were 60% for patients with a complete, major, or partial pathologic response and 18% for patients with no response.

**Figure 3:**
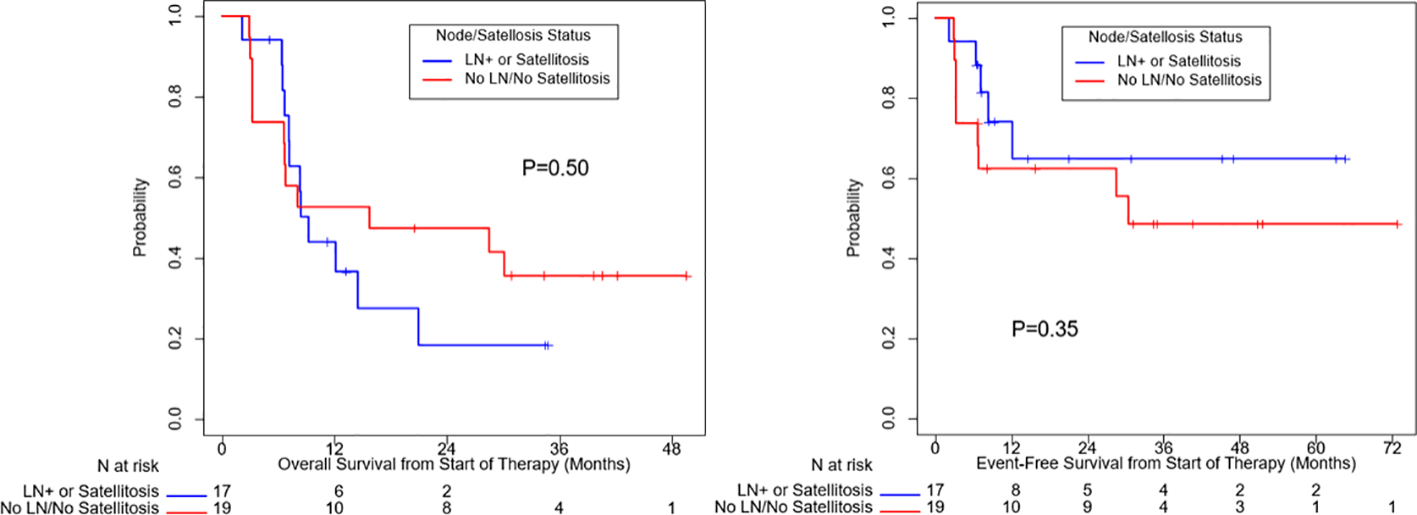
Overall survival and event free survival stratified based on regional nodal or satellite involvement and localized disease only.

**Figure 4:**
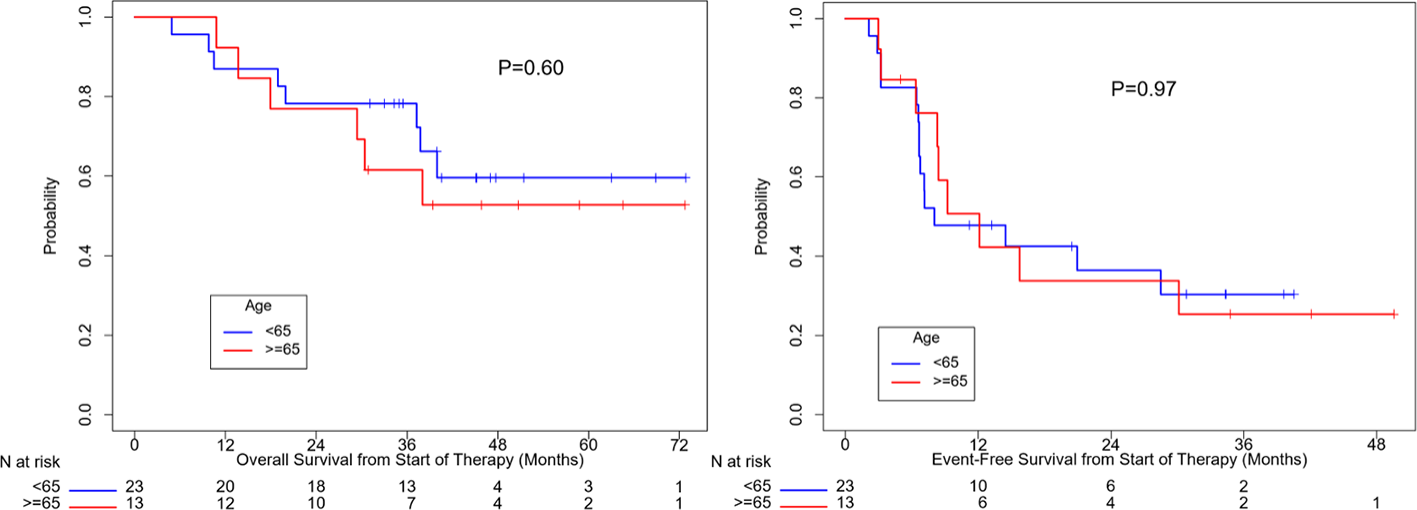
Overall survival and event free survival stratified based on age at diagnosis.

**Figure 5:**
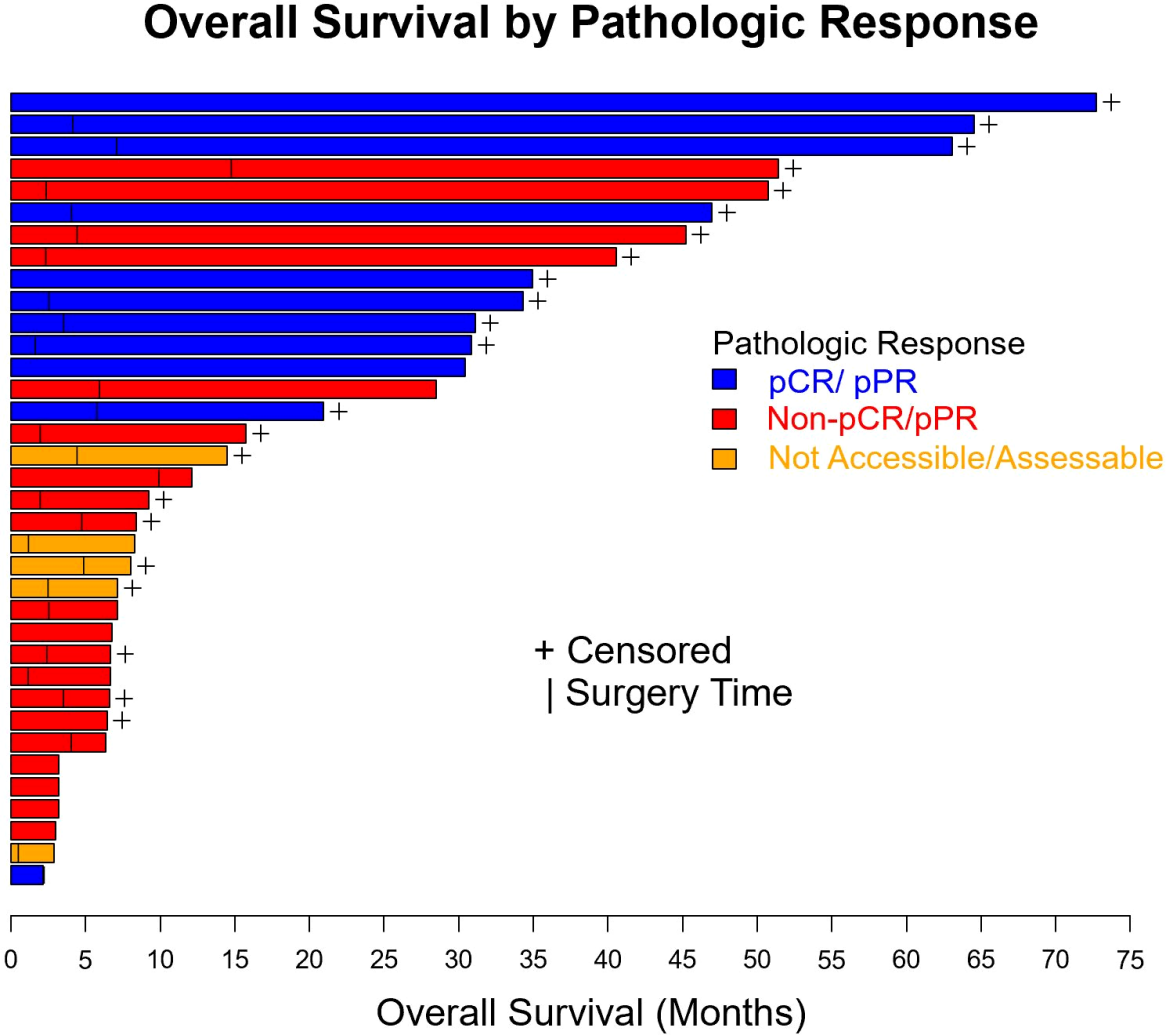
Swimmer plot of patients with resectable mucosal melanoma treated with neoadjuvant checkpoint inhibitors with pathologic response (blue), no pathologic response (red), and unknown pathologic response (orange). pCR, pathologic complete response, pPR, pathologic partial response.

**Figure 6:**
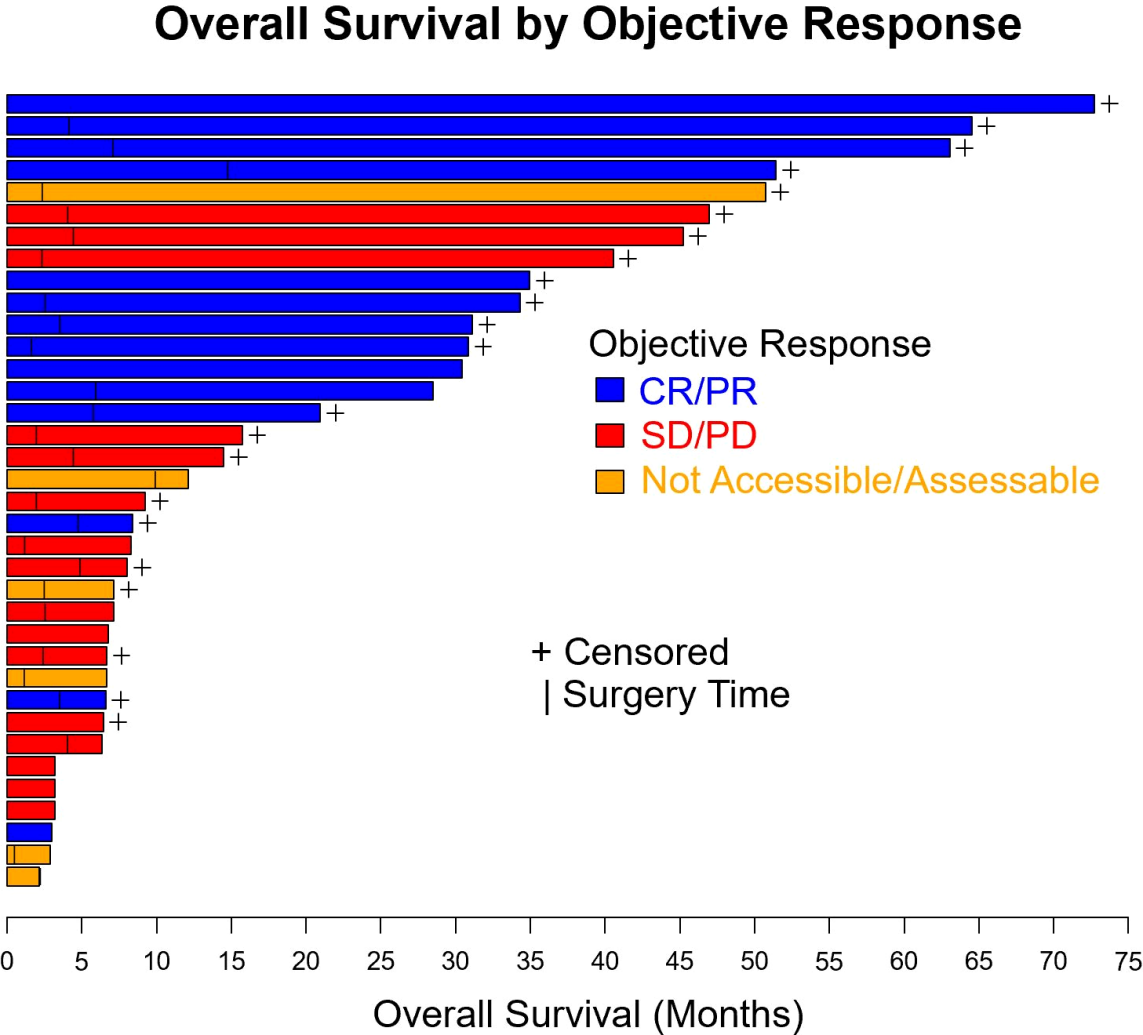
Swimmer plot of patients with resectable mucosal melanoma treated with neoadjuvant checkpoint inhibitors with objective response (blue), no objective response (red), and unknown objective response (orange). CR, complete response, PR, partial response, SD, stable disease, PD, progressive disease.

OS (p=0.10) and EFS (p=0.24) were higher for patients who received combination anti-PD1 and anti-CTLA4 compared to anti-PD1 or anti-CTLA4 monotherapy, but the difference was not significant (**Supplemental Figure 1**). There was no significant difference in OS (p=0.50) or EFS (p=0.35) between patients with localized disease only and with regional nodal or satellites disease (**Figure 3**). There was no significant difference in OS (p=0.60) or EFS (p=0.97) between patients age ≥ 65 or age < 65 (**Figure 4**). OS stratified by objective response and pathologic response were described in **Figures 5** and **6** and demonstrated improved OS for patients with an objective or pathologic response.

Fourteen (39%) patients had grade 3 or above toxicities (**Table 6**). The most common were transaminitis (14%), colitis (11%) and hypophysitis (11%). Among the 14 patients with grade 3 or above toxicities, 13 patients had combination Anti-PD1 and Anti-CTLA4 and 1 patient had Anti-PD1. Among the 6 patients who progressed to unresectable prior to surgery, only 1 patient had grade 3 or above toxicities.

**Table 6:** Toxicities per common terminology criteria for adverse events version 5.0 of patients with mucosal melanoma treated with neoadjuvant checkpoint inhibitors.

Total number of patients	36	%
Grade 3 or above toxicities	14	39
Endocrinologic AE:		
Hypophysitis	4	11
DM/DKA	1	3
Adrenal insufficiency	1	3
Gastrointestinal AE:		
Colitis	4	11
Transaminitis	5	14
Neurologic AE:		
Neuropathy	1	3
Optic neuritis	1	3
Other AE:		
Pneumonitis	1	3
Dermatitis	2	6
Pyrexia	1	3
Nephritis	1	3

AE, adverse events.

## Discussion

There is currently no standard of care for neoadjuvant or adjuvant systemic therapy for resectable MM. Patients treated with surgery alone for resectable MM have poor prognosis. RFS and OS with surgery alone have been reported to be around 5 and 21 months, respectively (11). Unlike cutaneous melanoma, MM does not have a standardized staging system such as that available for cutaneous melanoma by the American Joint Committee on Cancer 8^th^ edition to allow oncologists to understand prognosis and further risk stratify patients for adjuvant systemic treatments (17). MM patients have often been excluded or under-represented in landmark clinical trials that have led to FDA approval for adjuvant systemic therapies in cutaneous melanoma. For example, CHECKMATE 238 included 29 (3%) patients with MM and the subgroup analysis did not show better outcome with either adjuvant nivolumab or ipilimumab (18). KEYNOTE 054 excluded MM patients entirely (19). In a single center randomized phase II study, adjuvant chemotherapy with temozolomide and cisplatin improved OS and RFS compared to high dose interferon or surgery alone (11). However, adjuvant chemotherapy has not been the standard of care in practice as there is minimal evidence for efficacy in the metastatic setting (20). As adjuvant checkpoint inhibitors have shown benefits in resectable cutaneous melanoma, we believe that it is important to understand the outcome of patients with resectable MM who are treated with checkpoint inhibitors (18, 19). To our knowledge, this study is the first study to report the outcomes of MM patients who underwent treatment with neoadjuvant checkpoint inhibitors alone, showing a median EFS of 9.2 months and a median OS that had not been reached at a median follow up time of 37.9 months.

There is limited prospective data in metastatic MM patients treated with checkpoint inhibition, but based on a pooled analysis of MM subgroup across different clinical trials including CHECKMATE-067, we know MM has a lower response rate than cutaneous melanoma. While cutaneous melanoma patients have response rate of 58% and 44% to combination anti-PD1 and anti-CTLA4 or anti-PD1 monotherapy, respectively, MM patients have response rates of 37-43% and 30% to combination anti-PD1 and anti-CTLA4 and anti-PD1 monotherapy, respectively (21). MM patients in CHECKMATE-067 treated with combination anti-PD1 and anti-CTLA4 were found to have higher 5-year progression-free survival (PFS) and OS at 29 and 36% respectively, compared to patients treated with anti-PD1 monotherapy with 5-year PFS and OS of 14 and 17%, respectively (22). However, the role of combination immunotherapy in MM is controversial as a recent study suggests addition of anti-CTLA4 to anti-PD1 may not derive meaningful benefit for patients with MM in the metastatic setting (23). The retrospective cohort study of 545 patients with MM treated with anti-PD1 +/- anti-CTLA4 showed no significant difference in ORR, PFS or OS between patients treated with combination anti-PD1 and anti-CTLA4 and patients treated with Anti-PD1 alone. Nonetheless, as MM is seemingly less responsive to immunotherapy than cutaneous melanoma, patients with resectable MM may need addition of anti-CTLA4 to anti-PD1 to derive clinical benefit. Although no significant difference is observed in OS and EFS between patients who received neoadjuvant combination checkpoint inhibitors and single agent checkpoint inhibitor, this study is limited by low proportion (22%) of patients treated with anti-PD1 monotherapy and generally small sample size. Although not statistically significant, the median OS and median EFS are not reached vs. 8 months, and 15 months vs. 8 months respectively for patients who received neoadjuvant combination anti-CTLA4 and anti-PD1 compared to single agent anti-PD1/anti-CTLA4.

Use of neoadjuvant checkpoint inhibition can potentially downstage the tumor to facilitate resectability, improve the rate of R0 surgery and reduce the extent, and therefore, potential morbidity of surgery. Surgical resection remains the mainstay of treatment for resectable MM but surgical comorbidities are often more significant due to anatomical locations of disease (24). For example, abdominoperineal resection had been considered the surgical standard for anorectal MM. However, less invasive approaches such as wide local excision had shown to have similar survival outcomes compared to abdominoperineal resection (25, 26). By downstaging the tumor, neoadjuvant checkpoint inhibition may allow patients to receive less invasive surgery or eliminate the need for surgery. Three (8%) patients in this retrospective study did not undergo surgery due to complete response and the EFS and OS for these patients had not been reached. One of these three patients did pass away, but it was unrelated to melanoma.

Pathologic complete response had been used as a surrogate endpoint for different cancer types and led to approval of neoadjuvant treatment such as pertuzumab in HER2 positive breast cancer by the FDA (United States Food and Drug Administration) (27). Pooled analysis of cutaneous melanoma patients treated with neoadjuvant checkpoint inhibitors showed excellent outcomes for patients who had a complete or near complete pathologic response (28). Many hypotheses are currently being tested to investigate how pathologic responses translate to better clinical outcomes. Some of those hypotheses include exposure of immunotherapy-activated T cells to more neoantigens and early treatment of microscopic disease (29). In our retrospective review, we see that patients who had pathologic response had significantly improved OS and EFS. Patients who do not have a pathologic response to neoadjuvant checkpoint inhibitors have poor outcomes. By having an objective assessment of response to and tolerance of neoadjuvant checkpoint inhibitors, oncologists can help patients with suboptimal neoadjuvant treatment response make informed decisions for adjuvant therapy.

This study has limitations as a retrospective study. It is subjected to the effects of confounders and systemic errors such as information bias from missing data. Additionally, the small sample size is limiting; however, this is reflective of the rare nature of this malignancy. There is currently no standard of care for neoadjuvant or adjuvant systemic therapy for patients with resectable MM and the findings in this manuscript fulfills an unmet need in MM patient care.

In conclusion, this study is the first to characterize outcomes of resectable MM patients treated with neoadjuvant checkpoint inhibitors alone. In view of historic OS of less than 2 years with surgery alone, this study provides an update on the outcomes of resectable MM patients treated with a contemporary treatment paradigm and demonstrates a signal of efficacy for the use of neoadjuvant checkpoint inhibitors. This study supports further investigations using neoadjuvant immunotherapy.

## Data availability statement

The raw data supporting the conclusions of this article will be made available by the authors, without undue reservation, in compliance of institutional standard of MD Anderson Cancer Center, Houston, Texas as the data is stored in a secure server at MD Anderson Cancer Center, Houston, Texas.

## Ethics statement

Ethical review and approval was not required for the study on human participants in accordance with the local legislation and institutional requirements. Written informed consent for participation was not required for this study in accordance with the national legislation and the institutional requirements.

## Author contributions

JH, JM, RA contributed to conception and design of the study. RB, SFa, PN, and RA contributed monitoring and guidance as senior authors. RB performed the statistical analysis. SFa performed objective response assessment. PN performed pathologic response assessment. JH wrote the first draft of the manuscript except for the introduction. JM wrote the first draft of introduction. MT, MDW, AD, IG, JMc, SP, HT, MKW, SFi, EH, EK, MR, RW, SS, MF, LM, AJ, CP, AG, AB, DM, AF, SFa, PN contributed to clinical care of patients reviewed in this study. All authors contributed to manuscript revision, read, and approved in the submitted version. All authors contributed to the article and approved the submitted version.

## Conflict of interest

JH and RW were employed by MD Anderson Cancer Center. MDW, MAD, AD, IG, JMc, SP, HT, MKW, SFi, EH, EK, MR, SS, MF, LAM, AJ, CP, AG, AB, DM, AF, RB, SFa, PN and RA are employed by MD Anderson Cancer Center. MT is employed by University of California San Francisco. JM is employed by Hospital Moinhos de Vento. MAD is supported by the Dr. Miriam and Sheldon G. Adelson Medical Research Foundation, the AIM at Melanoma Foundation, the NIH/NCI 1 P50 CA221703-02, the American Cancer Society and the Melanoma Research Alliance, Cancer Fighters of Houston, the Anne and John Mendelsohn Chair for Cancer Research, and philanthropic contributions to the Melanoma Moon Shots Program of MD Anderson. MAD has been a consultant to Roche/Genentech, Array, Pfizer, Novartis, BMS, GSK, Sanofi-Aventis, Vaccinex, Apexigen, Eisai, Iovance, and ABM Therapeutics, and he has been the PI of research grants to MD Anderson by Roche/Genentech, GSK, Sanofi-Aventis, Merck, Myriad, Oncothyreon, ABM Therapeutics, and LEAD Pharma. MAD receives institutional clinical trial support outside the submitted work from Pfizer. LAM receives research funding from AstraZeneca for unrelated research. PN receives grant from Melanoma Research Alliance (# 570806). MW is a consultant and serves on scientific advisory board for Bayer. MF is a consultant for Stryker and Seagen and receives research funding from AkesoBio and GSK. SP reports: Institutional clinical trial support outside the submitted work from Bristol Myers Squibb, Foghorn Therapeutics, Ideaya, InxMed, Lyvgen, Novartis, Provectus Biopharmaceuticals, Seagen, Syntrix Bio, TriSalus Life Sciences and advisory board, steering committee, data safety monitoring board, and consulting fees outside the submitted work from: Advance Knowledge in Healthcare, Cardinal Health, Castle Biosciences, Delcath, Immunocore, Novartis, TriSalus Life Sciences, Bristol Myers Squibb. The handling editor RC declared a past co-authorship with the author SP. AJ has stock options from Avenge Bio and Green Fire Bio. He reports advisory fees from Macrogenics, GLG, Guidepoint, NuProbe, AvengeBio, Green Fire Bio, GI Innovation, Theolytics, Two XAR. He reports clinical trial funding to Institution from BMS, Merck, AstraZeneca, Iovance, Immatics, Aravive, Alaunos, Xencor, Avenge Bio, and Macrogenics. CP is a consultant for UptoDate Penile Cancer Series, Wolters Kluwer Publisher. IG receives research funding from BMS, MERCK, and Pfizer and serves in consulting roles or advisory board in BMS, Novartis, LEAL Therapeutics. RA receives research support from Novartis, Merck, Bristol-Myers Squibb, Iovance and Obsidian; consultancy for Novartis, Bristol-Myers Squibb and Iovance.

The remaining authors declare that the research was conducted in the absence of any commercial or financial relationships that could be construed as a potential conflict of interest.

## Publisher’s note

All claims expressed in this article are solely those of the authors and do not necessarily represent those of their affiliated organizations, or those of the publisher, the editors and the reviewers. Any product that may be evaluated in this article, or claim that may be made by its manufacturer, is not guaranteed or endorsed by the publisher.
