# Supplementary material for: Neoadjuvant checkpoint inhibitor immunotherapy for resectable mucosal melanoma
Source: Front Oncol. 2022 Oct 17;12:1001150. doi: 10.3389/fonc.2022.1001150 (PMC9618687; doi:10.3389/fonc.2022.1001150)

Supplementary Material

1 Supplementary Figures and Tables

1.1 Supplementary Tables

Supplementary Table 1: Neoadjuvant systemic therapy name, dose, frequency, and median number of cycles of patients with mucosal melanoma treated with neoadjuvant checkpoint inhibitors.

Total Number of Patients	36	%	Median number of cycles (range)
**Combination neoadjuvant Anti-CTLA4 and Anti-PD1**	28	78	
Ipilimumab 3mg/kg and Nivolumab 1mg/kg every 3 weeks	19	53	3 (1-4)
Ipilimumab 1mg/kg and Nivolumab 3mg/kg every 3 weeks	4	11	2.5 (2-3)
Not accessible	5	14	
**Anti-PD1 monotherapy**	7	19	
Nivolumab 240mg every 2 weeks	7	19	4 (4-13)
**Anti-CTLA4 monotherapy**	1	3	
Not accessible	1	3	4

Supplementary Table 2: Treatment received stratified by primary tumor site location.

**Treatment by sites of primary disease**	#
**Anorectal**	19
Underwent surgery	14
Adjuvant radiation	5
Adjuvant systemic therapy	12
No surgery due to progression	2
No surgery due to complete response	3
**Urogenital**	9
Underwent surgery	5
Adjuvant radiation	1
Adjuvant systemic therapy	3
No surgery due to progression	4
**Head and neck**	6
Underwent surgery	6
Adjuvant radiation	3
Adjuvant systemic therapy	1
**Esophageal**	2
Underwent surgery	2
Adjuvant radiation	0
Adjuvant systemic therapy	1

Supplementary Table 3: Reasons of patients who received neoadjuvant checkpoint inhibitor but did not undergo surgery. IrAE = immune related adverse events.

Number of patients who did not undergo surgery	9
Complete response prior to surgery	3
Local progression to unresectable disease prior to surgery	3
Distant organ progression prior to surgery	1
Grade ≥ 3 IrAE and then local progression to unresectable disease prior to surgery	1
Non-IrAE and then distant organ progression prior to surgery	1

Supplementary Table 4: Objective response, pathologic response, and adverse events during neoadjuvant therapy of patients who received adjuvant therapy. pCR = pathologic complete response; pPR = pathologic partial response; pNR = no pathologic response. CR = complete response; PR = partial response; SD = stable disease; PD = progressive disease.

Number of patients received adjuvant therapy	17
Pathologic response	
pCR or near pCR	6
pPR	2
pNR	6
Not accessible	3
Objective response	
CR	6
PR	2
SD	1
PD	4
Not accessible	4
Grade ≥ 3 adverse events during neoadjuvant therapy	5

1.2 Supplementary Figures

Supplementary Figure 1: Overall survival (1A) and event free survival (1B) stratified based on neoadjuvant checkpoint inhibitor regimen.

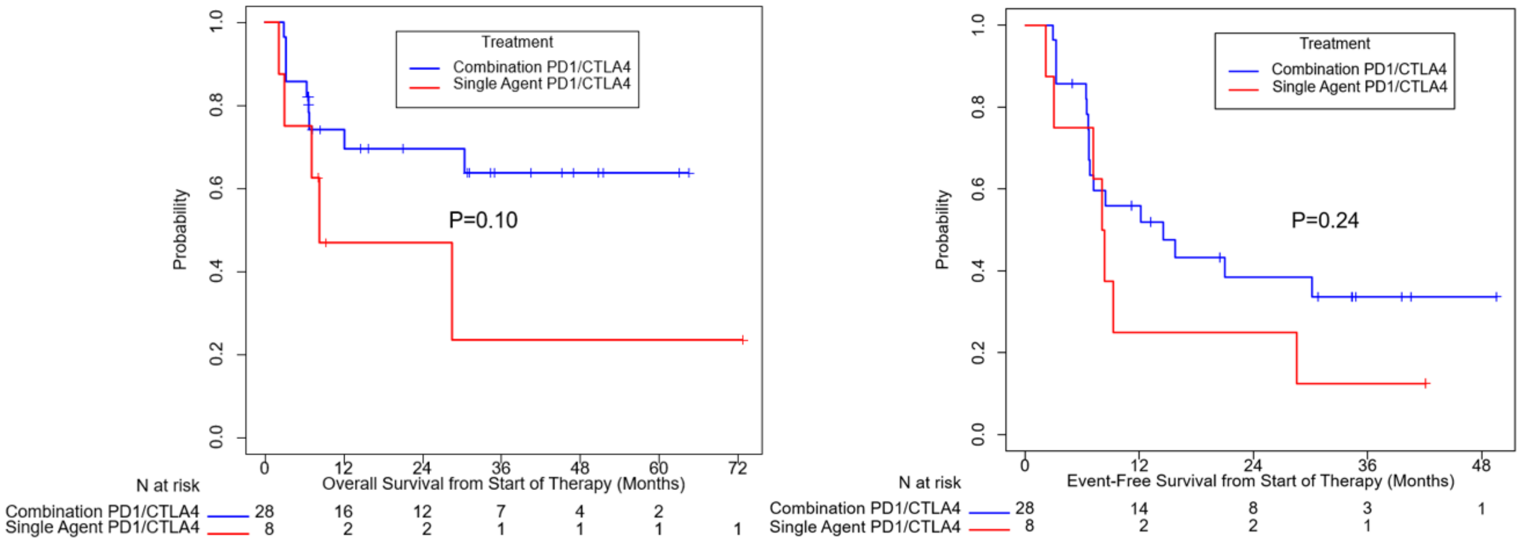

Supplementary Figure 2: Recurrence free survival of patients treated with neoadjuvant immunotherapy.

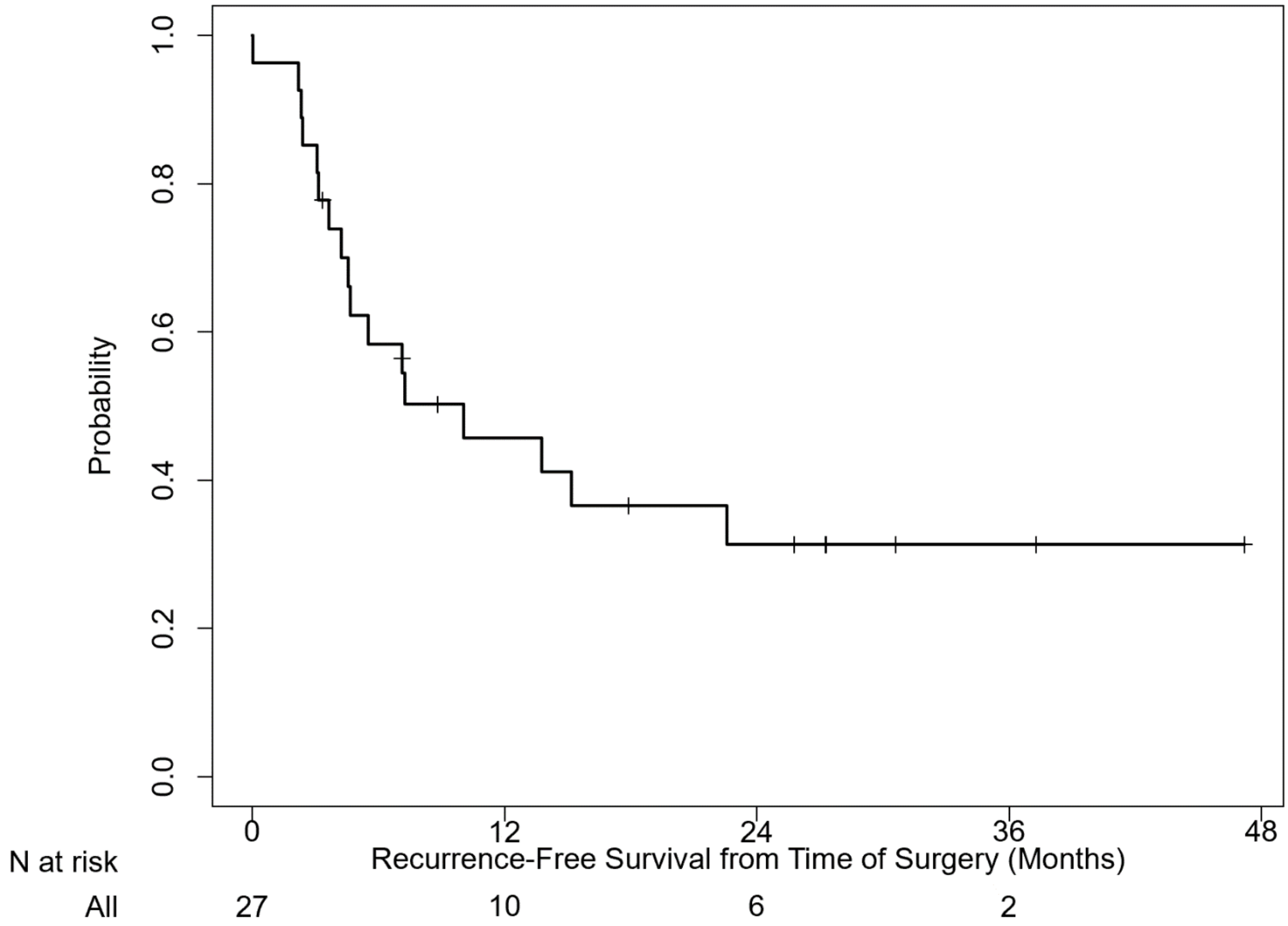

Supplement: Supplementary file 1 [file DataSheet_1.pdf]
